# Covalent inorganic complexes enabled zinc blende to wurtzite phase changes in CdSe nanoplatelets†

**DOI:** 10.1039/d3sc04296k

**Published:** 2023-11-08

**Authors:** Xinke Kong, Lin Ru, Junjun Ge, Yalei Deng, Pan-ke Zhang, Yuanyuan Wang

**Affiliations:** a State Key Laboratory of Coordination Chemistry, State Key Laboratory of Analytical Chemistry for Life Science, School of Chemistry and Chemical Engineering, Nanjing University Nanjing 210023 China wangyy@nju.edu.cn

## Abstract

Phase changes in colloidal semiconductor nanocrystals (NCs) are essential in material design and device applications. However, the transition pathways have yet to be sufficiently studied, and a better understanding of the underlying mechanisms is needed. In this work, a complete ligand-assisted phase transition from zinc blende (ZB) to wurtzite (WZ) is observed in CdSe nanoplatelets (NPLs). By monitoring with *in situ* absorption spectra along with electrospray ionization mass spectrometry (ESI-MS), we demonstrated that the transition process is a ligand-assisted covalent inorganic complex (CIC)-mediated phase transition pathway, which involves three steps, ligand exchange on ZB CdSe NPLs (Step 1), dissolution of NPLs to form CICs (Step 2), and conversion of CdSe–CIC assemblies to WZ CdSe NPLs (Step 3). In particular, CICs can be directly anisotropically grown to WZ CdSe NPL without other intermediates, following pseudo-first-order kinetics (*k*_obs_ = 9.17 × 10^−5^ s^−1^). Furthermore, we demonstrated that CICs are also present and play an essential role in the phase transition of ZnS NPLs from WZ to ZB structure. This study proposes a new crystal transformation pathway and elucidates a general phase-transition mechanism, facilitating precise functional nanomaterial design.

## Abstract 2

The pathway of covalent inorganic complex (CIC)-mediated phase transition from ZB CdSe NPLs to WZ CdSe NPLs.

## Graphical abstract

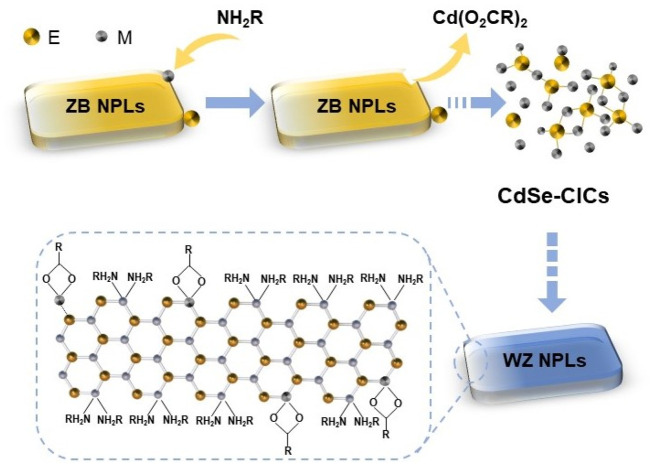

## Introduction

Colloidal semiconductor nanocrystals (NCs) have been the focus of worldwide interest over the past three decades, with applications ranging from biomedicine to energy and the environment.^1–3^ Their structures are defined by the periodic arrangement of repeating units, which determines the size, electronic energy band structure, and other physicochemical properties.^4–8^ From this point of view, an in-depth understanding of the crystalline phase, including the controlled preparation and phase transformation, is critical for the rational design of NCs.^9,10^

To date, advances in synthesis have enabled the experimentally precise preparation of various colloidal semiconductor NCs with well-defined crystalline arrangements, including zinc blende (ZB) or wurtzite (WZ) structure.^11–17^ However, studying transitions between crystal structures is more complex and still in its infancy, mainly because current studies usually focus on NCs grown by successive additions of precursors. As a result, slight differences in growth between individual crystals produce a statistical distribution in size and shape, leading to heterogeneity and complexity of the crystal surface. In contrast, the discrete growth of nanoplatelets (NPLs) in one dimension (thickness) leads to precise atomic layer thicknesses and well-defined crystal planes. In addition, NPLs with representative atomic arrangements have highly anisotropic shapes and large lateral dimensions, providing an ideal platform for studying phase transitions.^18–23^ For example, Pradhan *et al.* found a partial phase transformation from the WZ to the ZB structure in ZnS NPLs at high temperatures with Mn ions insertion.^24^ Yang *et al.* achieved the WZ to ZB phase transition of CdSe NPLs by simulating electron-beam.^25^ Recently, Peng *et al.* discovered a ligand-assisted crystal facet reconstruction behavior in CdSe/CdS core/shell NPLs.^26^ Although phase conversion of NPLs has been observed in various studies, the underlying mechanisms are still under debate.^24–27^ One possible reason is that UV absorption spectroscopy was usually used to monitor the transition process, and a featureless spectrum was considered to have no changes or no new material production in the reaction. Such subjective conclusions hindered the exploration of intermediates and their evolutionary pathways. Recent studies by Yu's group indicated that a particular substance was formed prior to the nucleation and growth of NCs, which was relatively transparent in optical absorption.^28–30^ This finding motivates us to re-examine the crystal transformation process.

**Scheme 1:**
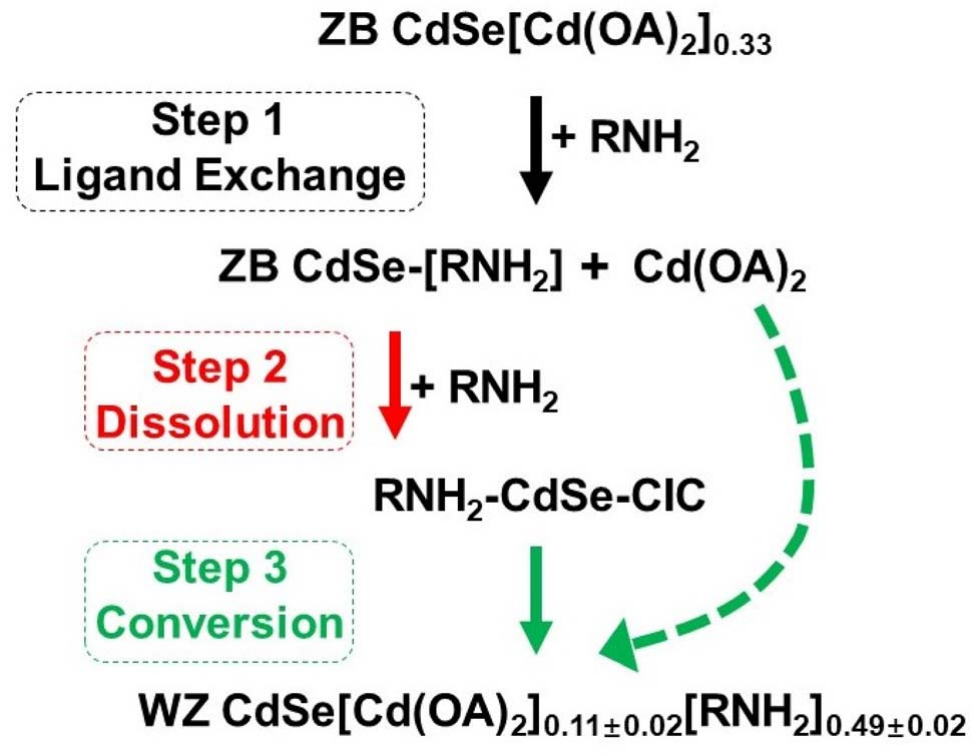
Schematic of the evolution mechanism proposed for the primary amine-driven phase transfer from ZB CdSe NPLs to WZ CdSe NPLs. In this process, three steps were involved: ligand exchange (Step 1), dissolution (Step 2) of NPLs, and conversion of CICs to WZ NPLs (Step 3). The particular type of intermediates (CICs) was found to be a vital link between ZB and WZ NPLs.

In this work, we used CdSe NPLs as model systems to study the evolutionary pathways of phase changes and to elucidate the transformation pathways and then extended the study to ZnS NPLs. We demonstrated covalent inorganic complexes (CICs) were important intermediates in the phase change process. It is worth noting that although CICs have been considered as important species for bridging molecules and clusters, their role in crystal phase transitions has yet to be studied.^28,29,31–33^ We showed that the process of phase evolution involved three steps (Scheme 1). In the first step, the primary amine as an L-type ligand replaced the X-type ligand (oleate, OA) on the NPL surface in the form of cadmium oleate (Cd(OA)_2_) through a ligand exchange process, tuning nonstoichiometric ZB CdSe NPLs to stoichiometric ones (Step 1), which were further etched from the edge to form CICs with covalent Cd–Se bonds (Step 2). Then, subsequent nucleation and anisotropic growth of CdSe–CICs at elevated temperature allowed the direct formation of WZ CdSe NPLs, and the Cd(OA)_2_ from Step 1 further functioned as a Z-type ligand to stabilize NPLs in nonpolar solvents (Step 3).

Further studies showed that CIC is a new types of precursor that can be directly anisotropically assembled into WZ CdSe NPL without going through magic size clusters (MSCs), following pseudo-first-order kinetics with a rate constant of *k*_obs_ = 9.17 × 10^−5^ s^−1^ (*t*_1/2_ = 2.1 h). In addition, we re-evaluated the Mn-doping-induced crystal phase transition from WZ to ZB structure of Zn-based NPLs. We found the presence of a CIC-mediated process, demonstrating the generality of the mechanism in explaining other phase transition processes. This study not only achieved the complete phase changes from the ZB to WZ structure in NPLs but also provided an in-depth understanding of the CIC-mediated phase evolution process in NCs, pointing to a new direction for the rational design of functional nanomaterials.

## Results and discussion

**Fig. 1:**
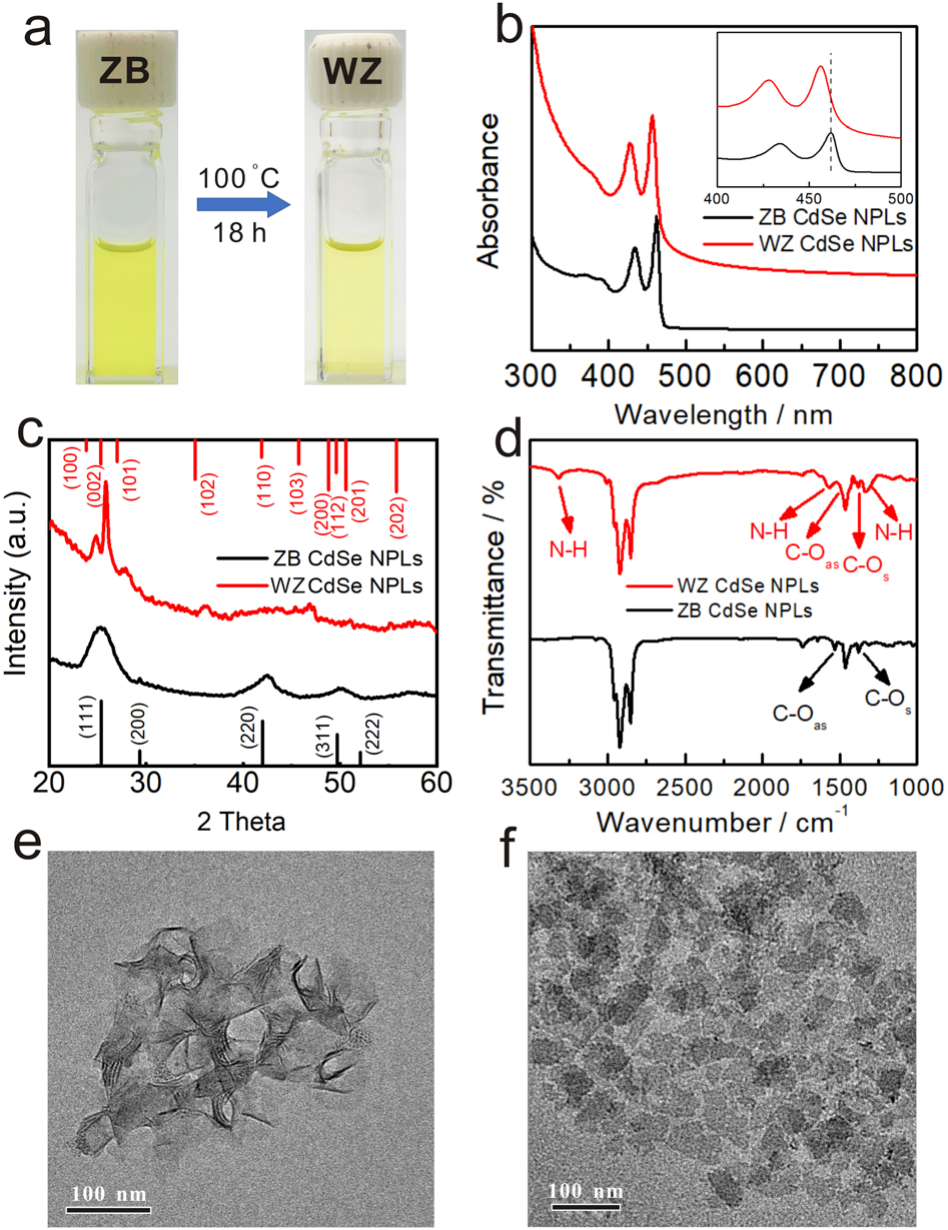
The comparison of CdSe NPLs before (ZB) and after (WZ) phase transformation in the octylamine system. (a) Reaction condition and photographs of source material ZB CdSe NPLs in toluene (left) and product WZ CdSe NPLs in toluene (right). (b) UV-vis absorption spectra, (c) XRD patterns, and (d) FT-IR spectra of NPLs before (ZB, black line) and after (WZ, red line) the conversion. TEM images of (e) as-synthesized ZB CdSe NPLs and (f) converted WZ CdSe NPLs.

The OA-capped ZB CdSe NPLs with three and half monolayers (3.5 MLs) were synthesized and stabilized in toluene (Fig. 1a left) according to previously reported procedures.^34^ The UV-vis spectrum showed three characteristic absorption peaks at 462, 434, and 392 nm, corresponding to hh1–e1 (the first transition between the heavy atom and the electron), lh1–e1 (the first transition between the light atom and the electron) and so–e (light-hole–heavy-hole splitting transitions) of the NPLs (Fig. 1b black). The stoichiometry of NPLs was determined to be CdSe[Cd(OA)_2_]_0.33_ by inductively coupled plasma optical emission spectroscopy (ICP-OES). A pure and well-defined ZB crystal structure was revealed by X-ray diffraction (XRD) characterization (Fig. 1c black), and the morphology of ZB CdSe NPLs with a large curled two-dimensional morphology was determined by TEM (Fig. 1e and S1a†).

### Observation of phase changes from ZB to WZ in CdSe NPLs

The transformation was performed by incubating the OA-capped ZB CdSe NPLs in pure *n*-octylamine (OCA) at 100 °C, after which a color change of the colloidal dispersion to pale yellow was observed (Fig. 1a, right). The products were collected as yellow solids after high-speed centrifugation.

TEM images showed the morphology of rectangular-shaped NPLs. The products retained the two-dimensional structure with an average width of 28 ± 5 nm and a length of 60 ± 8 nm (Fig. 1f and S1b†). Interestingly, all NPLs were exfoliated and lay flat on the copper grid, which differed from the former large curled shape (Fig. 1e). The UV-Vis spectra of the pale yellow precipitate dispersed in toluene exhibited nearly identical spectra corresponding to 1D quantum-confinement systems (Fig. 1b red).^35,36^ The absorption showed three characteristic features at 456 nm, 427 nm, and 378 nm, which were assigned to the hh1–e1, lh1–e1, and so–e. We noticed a slight scattering tail at longer wavelengths and attributed this to the partially bundled nature of the NPLs.^37,38^ Compared to the absorption spectrum of ZB CdSe NPLs (Fig. 1b black), the characteristic feature of converted CdSe NPLs was blue-shifted by 6 nm. In addition, we compared the spectra of the converted CdSe NPLs to the WZ CdSe NPLs synthesized by the conventional method (the reaction of cadmium acetate and selenourea in primary amine solution) and observed a 6 nm red-shift (Fig. S2a† black).

The XRD pattern of isolated solids indicated the crystal structure changes before and after incubation. As shown in Fig. 1c, the features of (111), (220), and (311), corresponding to the ZB crystal structure, completely disappeared (black line), while new peaks belonging to the (100), (101), (102) and (103) crystal planes of WZ-CdSe emerged at 24.8°, 27.8°, 36.1° and 46.8° (red line). It is noteworthy that the diffraction peaks of the WZ products were slightly shifted to larger angles with respect to the bulk lattice, indicating the contraction of the WZ lattice due to the lattice reconstruction caused by the compressive stresses applied by organic molecules on the WZ_(110)_ surface.^22,39^ Since the ZB and WZ NPLs have different electronic band structures in the center of the Brillouin zone, we speculated that the blue-shift, as mentioned above in the absorption spectra, was related to the crystal structure.^40^ The combination of TEM images, UV-vis spectra, and XRD measurements demonstrated that CdSe NPLs achieved a complete phase transformation from ZB to WZ in an OCA system and maintained their good two-dimensional structure.

To investigate the surface environment, we first used FT-IR spectroscopy to study the binding of ligands to the surface of the obtained WZ CdSe NPLs. As shown in Fig. 1d, in addition to the pronounced N–H stretching (3318.4 cm^−1^), little C=O stretching was observed, indicating a small number of carboxyl groups on the surface. In addition, the Cd : Se ratio of the product (∼1.11 : 1) was higher than that of conventional WZ CdSe NPLs (∼1 : 1), indicating that excess Cd was attached to the surface. The average coverage of ligands on the obtained WZ CdSe NPL surface was further studied by thermogravimetric analysis (TGA). Approximately 19.96% of the weight loss before 350 °C was attributed to the dissociation of surface OCA ligands (Fig. S2d†), consistent with conventional WZ NPLs (Fig. S2c†). In addition, a small amount of OA branch attachment (Fig. S2d† blue line) resulted in further weight loss between 350 and 500 °C.^41,42^ The combination of ICP-OES and TGA established that the WZ CdSe NPLs obtained from the conversion of ZB NPLs in primary amine have the molecular formula CdSe[Cd(OA)_2_]_0.11±0.02_[R–NH_2_]_0.49±0.02_. Therefore, we speculated that in addition to –NH_2_, Cd(OA)_2_ may act as Z-type ligands to bind to the surface of NPLs.^43^ The diversity and complexity of the surface ligands of the converted CdSe NPLs may be one of the reasons for the 6 nm red-shift of converted NPLs compared to that of the conventional amine capped NPLs.^23,43–45^

To further verify the hypothesis, we added different amounts of Cd(OA)_2_ to the conventional WZ CdSe NPLs and used UV-vis spectra to monitor the evolution. The characteristic features were gradually red-shifted with increasing amounts of Cd(OA)_2_ and finally stabilized at ∼473 nm (Fig. S2a†), consistent with a previous report.^43^ The relationship between the absorption position of NPLs (wavelength of hh1–e1 on the vertical axis) and Cd/Se ratio (on the horizontal axis) was plotted in Fig. S2b.† We found that when the absorption of hh1–e1 was at 456 nm, the Cd/Se ratio of the corresponding samples was approximately 1.11, which exactly matched the value of WZ CdSe NPLs obtained by phase transformation from ZB CdSe NPLs. The results obtained from optical and surface state measurements demonstrated that the 6 nm red-shift in the spectrum of the converted NPLs compared to that of conventional WZ NPLs was associated with the NPL surface environment rather than the crystal surface tension, suggesting that the ligands of NPLs can tune the transition energy and extinction coefficient of the exciton band (details were discussed in the following section).^45^

To fully understand and explore the mechanisms during the reaction, we monitored the phase transition process using UV-vis, FT-IR, XRD, TEM, and electrospray ionization mass spectrometry (ESI-MS). We found that covalent inorganic complexes (CICs) were vital reaction intermediates in the phase transition. In addition, we speculated that NPLs may have undergone three processes during the phase transition: ligand exchange (Step 1), dissolution (Step 2), and conversion (Step 3).

### Amine-driven ligand exchange on ZB CdSe NPLs (Step 1)

The red-shift and weakened intensity of the NPLs spectra suggest that ligand exchange and dissolution occurred at the initial reaction at high temperatures (Fig. S3a†). To separate these two steps and reveal the evolution of the ligand exchange process on NPLs, we decreased the reaction temperature (60 °C) to slow the reaction rate and monitored absorption changes using XRD measurement and *in situ* UV spectroscopy.

**Fig. 2:**
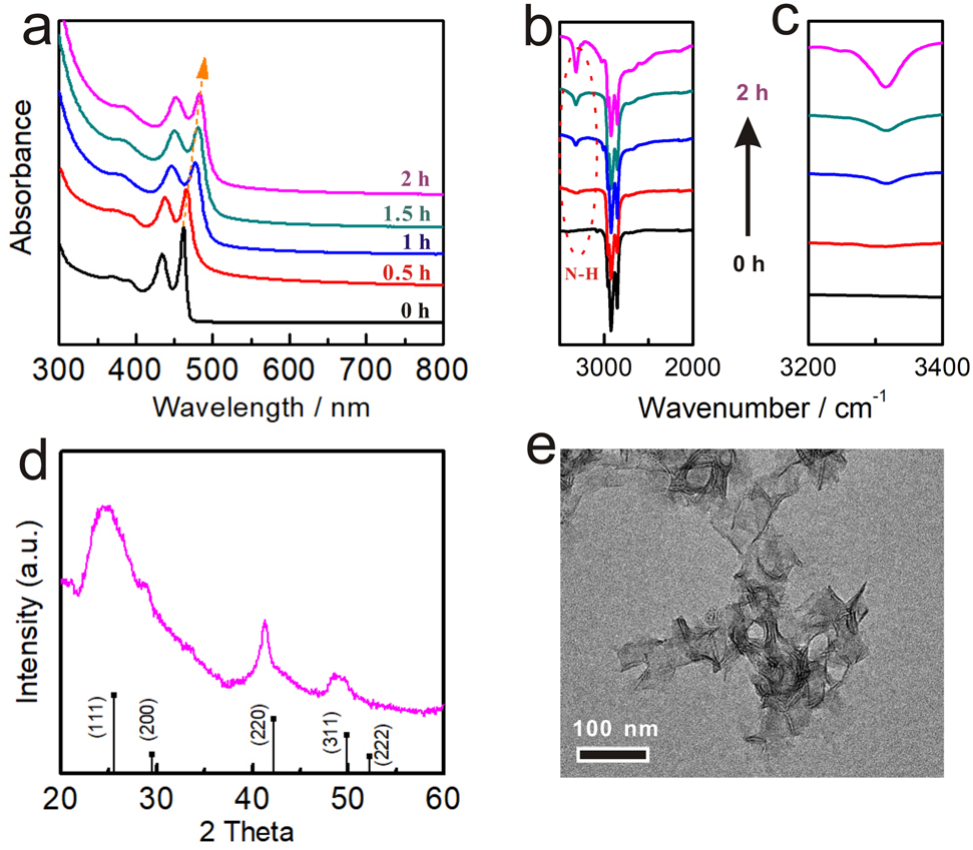
The evolution of ZB CdSe NPLs in Step 1 at 60 °C which was monitored by (a) UV-vis absorption spectra and (b and c) FT-IR. (d) XRD pattern and (e) TEM image of the ZB CdSe NPLs extracted from the reaction at 6 °C for 2 h.

The XRD analysis was used to examine the crystal structures of NPLs in this step, and the results confirmed that the ZB structure remained intact (Fig. 2d). In addition, the TEM images showed the overall retention of the large curled morphologies without any apparent damage (Fig. 2e), further demonstrating that no structural changes were involved in Step 1. We also performed the reaction at even lower temperatures (25 °C). As expected, the spectra of NPLs also only red-shifted (the hh1–e1 position to ∼478 nm) without any decrease in intensity (Fig. S4a†), and the morphology of NPLs was completely preserved (Fig. S4b†).

With increasing incubation time, the hh1–e1 absorption of ZB CdSe NPLs gradually red-shifted to ∼482 nm within the initial 2 h without any significant intensity decreased (Fig. 2a). We attributed the difference to the exchange of ligands on the surface of NPLs. FT-IR spectra showed a gradual increase in the intensity of the N–H stretch (3318.4 cm^−1^) from the OCA and a gradual decrease in the intensity of the C=O (1650.3 cm^−1^) from the OA as the reaction progressed, indicating that the substitution of X-type ligand (OA) by L-type ligand (OCA) (Fig. 2b and c). ^1^H NMR spectroscopy provided further evidence of the ligand exchange process. The chemical shift of the double-bonded protons of OA was used to distinguish between free and surface-bound molecules.^46,47^ Fig. S5a† shows an upfield shift ^1^H signal of cadmium oleate (5.33 ppm) *versus* free oleic acid. A corresponding upfield signal (5.26 ppm) was also detected in the supernatant after the reaction, indicating the occurrence of a ligand exchange reaction, where the difference in the degree of displacement was due to dielectric changes caused by solvent effects (Fig. S5b†).^46^ In addition to reaction temperature, solvents can also affect the reaction rate. For example, we annealed the ZB CdSe NPLs at 100 °C in a mixture of OCA/ODE (v : v = 1 : 2). The UV-vis absorption spectrum of NPLs after the reaction in Fig. S6a† was only red-shifted (to ∼482 nm) with no significant decrease in intensity. XRD pattern further indicated the integrity of the ZB crystal morphology (Fig. S6b†). These temperature and solvent control results supported our suggestion that ligand exchange was involved in Step 1.

It is worth noting that the stripping of OA was accompanied by removing cadmium atoms from the NPL surface, which decreased the NPL thickness and resulted in a blue shift in the UV absorption spectrum. However, we did observe a red-shift in the spectrum, similar to that observed in the ligand exchange reaction of ZB CdSe NPLs with ethylenediamine.^44^ We attributed the spectral shift to lattice stretching and an increase in the effective confinement dimension due to the electronic coupling between the surface ligands and the NPLs.^48–50^ This hypothesis was supported by the high-angle XRD measurements of the products at different incubation times (Fig. S7a†). In particular, the (002) crystal plane of ZB CdSe NPLs was split into two diffraction peaks, including the lateral (yellow line) and thickness (green line) directions.^48,51^ As the experiment progressed, there was a reduction of the diffraction angle along the vertical confinement (indicated by the green line) from 42.8° to 40.6°, suggesting an extension of the lattice.^49^ This also led to broadening of the diffraction peak. The Bragg equation was utilized to calculate the interplanar spacing with the respective lattice parameters as presented in Table S1.† The width decreased from 0.219 to 0.215 nm, whereas the thickness direction increased from 0.211 to 0.222 nm. Subsequently, the energy shift of 109.4 meV caused by a lattice strain change was calculated from eqn (S1).† ^52–54^ After being treated for an hour, the ZB NPLs' hh1–e1, which were capped with oleic acid ligands (2.683 eV), had a redshift to 2.573 eV (Δ*E* = 110.0 meV). Thus, the contribution of lattice expansion to the overall energy transfer was comparable. In addition, we noticed that the ligand exchange process was reversible. The addition of Cd(OA)_2_ to *n*-octylamine-capped NPLs shifted the absorption spectrum to its original position (Fig. S7b†).^23^

### Detection of CICs from the dissociation of ZB CdSe NPLs (Step 2)

**Fig. 3:**
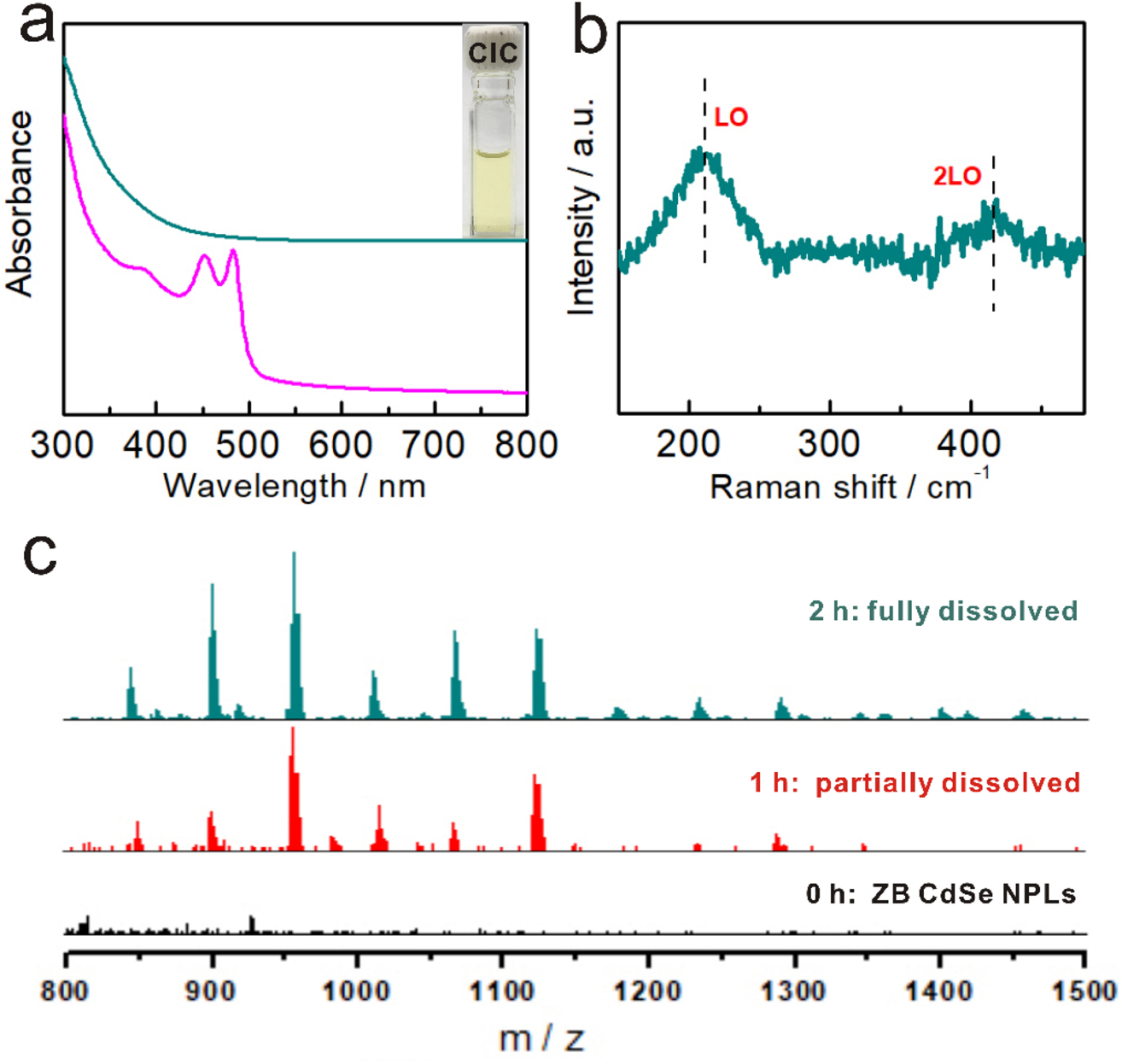
The evolution of ZB CdSe NPLs in Step 2. (a) UV-vis absorption spectra of ZB CdSe NPLs (before complete dissolution, pink) and CdSe–CICs (cyan). (b) Photograph (a, insert) and Raman spectra of CdSe–CICs in OCA. (c) ESI-MS spectra of the species detected for the samples extracted from the reaction at 100 °C for different times in the *m*/*z* range from 800 to 1500 Da.

As the reaction proceeded, the dispersion of NPLs gradually changed to a transparent solution (Fig. 3a inset), and the corresponding UV-Vis absorption spectrum became featureless (Fig. 3a cyan and S9a†), inicating the dissolution of the ZB CdSe NPLs (Step 2).

Investigating the dissolution products will help us better understand the specific reaction form at this step. Previous studies have shown that the decomposition of WZ NPLs tended to produce magic size clusters (MSCs) with characteristic absorption spectra.^38^ The formation of MSCs was ruled out by the absence of characteristic peaks in the UV-Vis absorption spectrum (Fig. 3a cyan). Further Raman spectroscopy revealed that the compounds present in the product solution were not ionic but in the form of Cd–Se covalent bonds with two characteristic peaks at 210 cm^−1^ (longitudinal optical, LO) and 415 cm^−1^ (2LO) (Fig. 3b). The covalent compound without characteristic absorption was very similar to the CICs proposed by Yu *et al.*^28^ In addition, transparent solutions also provided the prerequisites for ESI-MS studies. Fig. 3c shows the corresponding ESI-MS spectra (in the *m*/*z* range from 800 to 1500) of the samples from the reaction at different times. No signals were detected at the initial stage after the dispersion of ZB CdSe NPLs in *n*-octylamine (Step 1, corresponding to Fig. 3c, black), indicating that the NPLs remained intact and undissolved. When the reaction entered Step 2, multiple peaks corresponding to Cd–Se complexes appeared in the ESI-MS spectra, indicating the generation of CICs composed of Cd and Se isotopes (Fig. 3c, red). In addition, the intensity of these peaks gradually increased as the reaction proceeded (Fig. 3c, cyan). By comparing with the corresponding absorption spectra, we found that the intensities of the ESI-MS peaks gradually increased with decreasing absorption intensity and were highest when the UV-Vis absorption peak was featureless (Fig. 3c and S3a†). From the different predominant peaks detected within the ESI-MS spectra, the mass/charge ratio (*m*/*z*) ranged from 800 to 1500, indicating the presence of the 800 Da to 1500 Da fraction in CICs, similar to that reported by Yu *et al.*^55–57^ It should be noted that while ESI-MS can verify the existence of small covalent compounds, it cannot provide an exact identification of CICs composition due to the uncertainties in the distribution of ligands and charge.^58,59^ Although more detailed studies will be required in the future to make precise measurements of the composition of CIC, these results provided the first insight into the dissolution products of ZB NPLs in the form of covalently bound small molecular CICs.

**Scheme 2:**
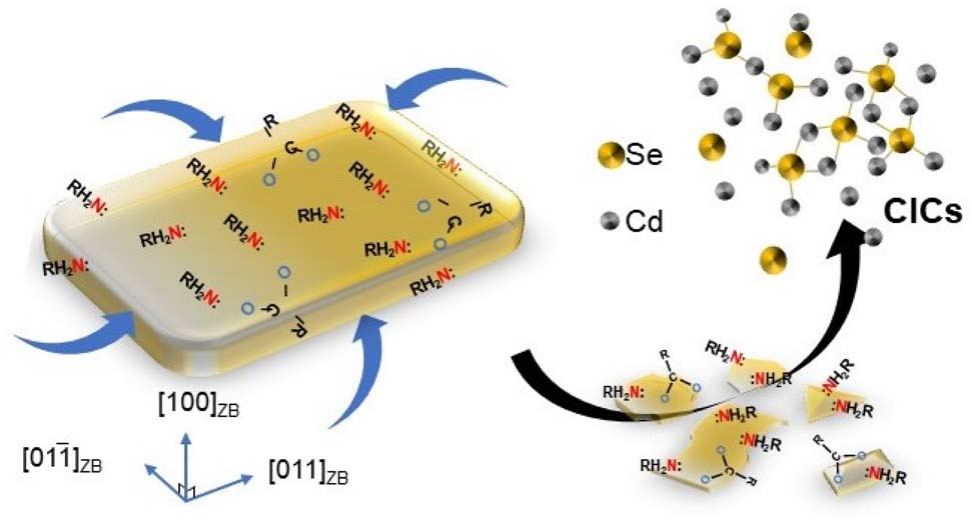
Schematic drawing illustrated the dissolution of ZB CdSe NPLs to CdSe–CIC under fatty primary amine attack and without other photo-detectable intermediates.

To further visualize the evolution process, we sought to monitor the crystallinity of the products at different reaction times. Fig. S8d† shows that the intensity of the XRD diffraction peak gradually decreased until it disappeared, indicating that the ZB NPLs were gradually etched. In addition, we monitored the morphological and dimensional changes of NPLs using TEM and found that NPLs did have gradual dissolution behavior. Specifically, the ZB CdSe NPLs with pseudo-tetragonal morphology gradually transformed into irregularly shaped flakes and quasi-particles of different sizes in the pure amine system and finally dissolved completely (Fig. S8a–c,† corresponding to Fig. S3a and b†). Interestingly, the ZB NPLs showed no significant perforation behavior on their (100)_ZB_ planes, and the thickness direction remained constant. These results indicated that etching occurs mainly at the periphery and at the edges of the circumferential surfaces of the ZB NPLs rather than at the (100)_ZB_ surface (as shown in Scheme 2), which was mainly because unsaturated Cd atoms near the edges and corners of the ZB NPLs, as well as the ridge positions at the boundaries, are more susceptible to amine attack, leading to the dissolution behavior.^22,60^

We, therefore, proposed that the attack of the metal site (M sites) by primary amines as L-type ligands resulted in the progressive dissolution of NPLs. In addition, we repeated the experiment using a tri-*n*-butylphosphine (TBP) system instead of the primary amine to incubate ZB CdSe NPLs. TBP is a Z-type ligand that binds to the E site and therefore does not lead to lattice dissolution and transformation.^46^ As expected, only a significant red shift (Fig. S10a,† hh1–e1 to 482 nm) was observed, with no apparent etching phenomenon in the ESI-MS spectra (Fig. S10c,† next paragraph for details). XRD characterization also confirmed that the product remains intact as a ZB structure (Fig. S10b†).

To illustrate the process, we draw a schematic diagram showing the role of ligands in the phase transition (Scheme S1†). The attack of the M site by primary amines (L-type ligand) led to the release of Cd(OA)_2_ (Step 1) and the progressive dissolution of NPLs by forming CdSe–CICs (Step 2). Subsequently, CdSe–CICs were directly assembled and anisotropically grown into WZ CdSe NPLs with amine template-assisted, after which the free cadmium oleate was attached to the E site as a Z-type ligand to passivate the NPLs (Step 3 and see below).

### Conversion of CdSe CIC assemblies to WZ CdSe NPLs (Step 3)

**Fig. 4:**
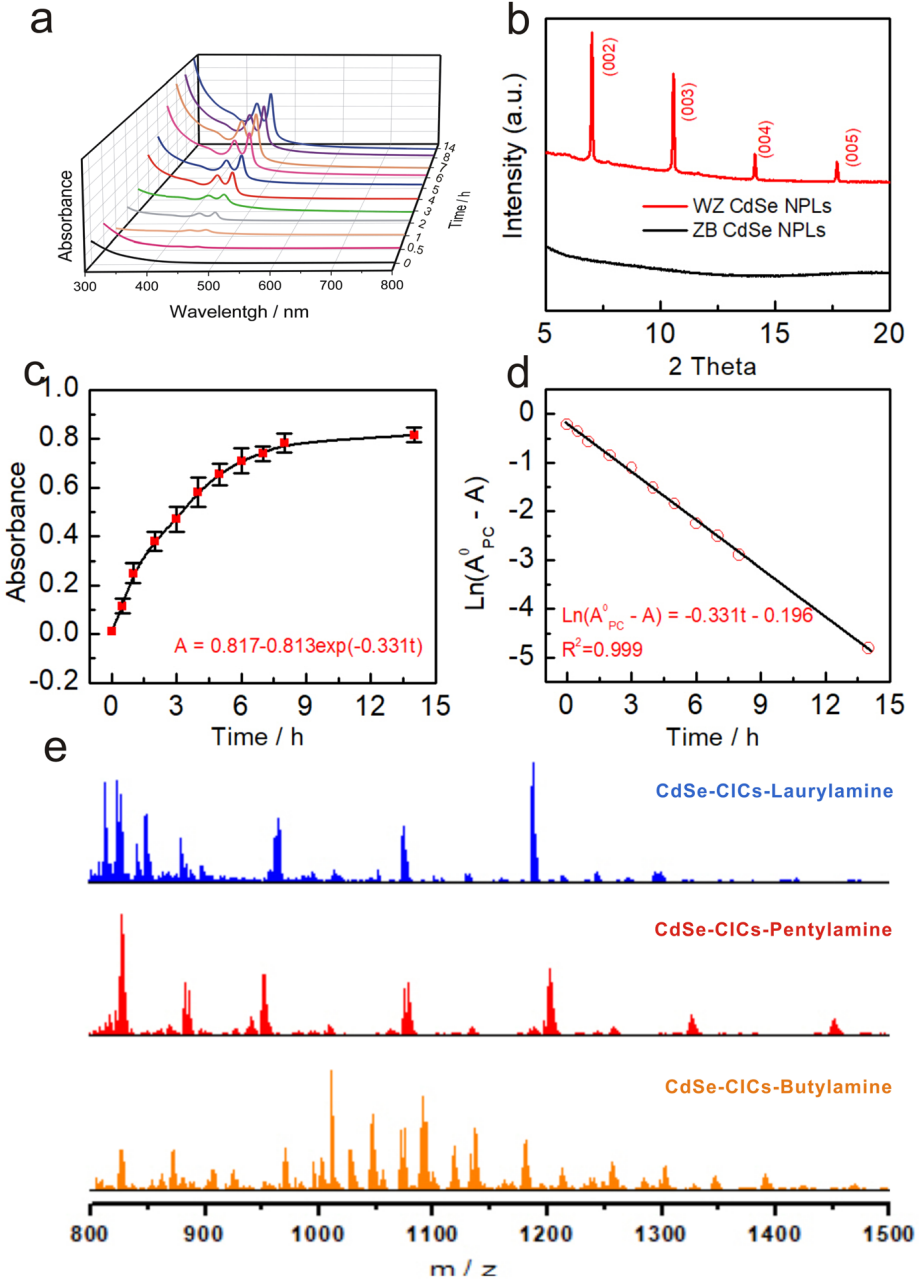
The evolution of CdSe CICs in step 3 at 100 °C, in which WZ CdSe NPLs were formed (a–d). (a) UV-vis absorption spectroscopy study of the evolution of WZ CdSe NPLs from a reaction mixture, in which the initial reaction time in Step 3 corresponded to the 10th hour of the entire reaction process (from Step 1). (b) Low-angle XRD patterns of the transformation before (ZB, black line, without template diffraction) and after (WZ, red line, with octylamine template diffraction). (c) Time-dependent absorbance at 456 nm (with the deduction of the background absorbance) was collected from the eleven dispersions and fitted by first-order equation (*A* = *A*^0^ − *A*^0^exp(−*k*_1_*t*)). (d) Time-dependent ln(*A*^0^ − *A*) with *A*^0^_CIC_ value was 0.817 in (a) 14 h. The corresponding linear fitting line is based on the first-order equation (ln(*A*^0^ − *A*) = −*k*_1_*t* + *I*). (e) ESI-MS spectra of the intermediate CdSe–CIC under different primary amine systems in the *m*/*z* range from 800 to 1500 Da.

Further incubation of CICs in primary amine brought the reaction to the third step (Step 3), where the featureless absorption was replaced by two absorption features at 456 nm and 427 nm. As discussed above, these features can be assigned to the hh1–e1 and lh1–e1 of WZ CdSe NPLs (Fig. S9b,† red curve). *In situ* absorption spectrometry monitored the evolution of CdSe CICs in *n*-octylamine. No other intermediates (*e.g.*, clusters) were observed (Fig. 4a), suggesting a direct conversion pathway from CICs to NPLs. In Fig. S9d,† the Raman spectrum showed that the CdSe LO phonon was identified with a stronger signal at 210 cm^−1^, which was much stronger than that of CICs, indicating better crystal structures.^61^ As XRD (Fig. 1c, red) and TEM (Fig. 1f) further confirmed, the NPLs had better crystallinity and were pure WZ phase. We need to emphasize that the morphology and lateral dimensions of the transformed WZ NPLs are not directly related to the dimensions of the original ZB NPLs but rather to the lamellar templates during the transformation of CICs to NPLs (Step 3). As evidenced by Fig. S13,† the morphology and lateral dimensions of the product WZ NPLs can be modulated by using different amine templates, which was similar to the case of the conversion from magic-size clusters (MSCs) to NPLs.^36,62,63^

The low-angle XRD diffraction patterns demonstrated the structures of NPLs as lamellar, amine-bilayer mesophases with a *d* spacing of 2.62 ± 0.02 nm (Fig. 4b), which was consistent with the number previously demonstrated by Buhro and co-workers.^35,37,38^ Interestingly, the lamellar structure was also present in the CIC assemblies with a *d* spacing of 2.37 ± 0.02 nm (Fig. S9c†), similar to that of a metal salt template (2.25–2.61 nm) and slightly smaller than the MSCs, probably due to the smaller size of the CICs compared to the MSCs.^35^ To eliminate the possibility that the template was formed by self-assembly during the precipitation process, we performed solution XRD, which demonstrated that the templates originated from the CICs (Fig. S9c,† red trace). ESI-MS measurements showed that the amount of CICs gradually decreased with the production of WZ CdSe NPLs in Step 3 until they completely disappeared, further demonstrating the direct conversion pathway from CICs to NPLs (Fig. S11e†). Therefore, we concluded that CICs spontaneously assemble into lamellar structures with the help of primary amines and then convert directly to NPLs without forming clusters as intermediates.

In addition, we further investigated the effect of the carbon chain length of primary amines (C4 to C18) on the reaction process. As expected, the ZB CdSe NPLs in the primary amine systems followed the CIC-mediated mechanism to complete the crystal phase transition reaction (Fig. S11–S14†). Interestingly, CICs with different atomic number compositions were obtained in various amines (Fig. 4e and S12c†), but this does not affect the conversion to WZ NPLs (see ESI† for detailed discussion).

Previous studies have shown that WZ NPLs were usually prepared from MSCs.^32,33,39,40^ Here, we found a new and different pathway whereby CICs rather than MSCs can be used as intermediates to directly form WZ NPLs. We further investigated the evolutionary kinetics of the WZ NPLs growth process by monitoring the changes in absorbance at 456 nm (Fig. 4a) during the conversion of CdSe CIC assemblies to WZ CdSe NPLs in *n*-octylamine (Fig. 4c and d). The appearance of WZ CdSe NPLs followed pseudo-first-order kinetics with *k*_obs_ of 9.19 × 10^−5^ s^−1^ and *t*_1/2_ of 2.10 h (Fig. 4d). Notably, this rate constant was two times faster than the rate constant determined by Buhro *et al.* for the growth of MSCs to NPLs (*k*_obs_ = 5.14 × 10^−5^ s^−1^ (*t*_1/2_ = 3.8 h)), suggesting a higher reactivity of the CICs.^63^ However, this rate constant was slower than that of the conversion from CICs to MSCs determined by Yu *et al.* (*k*_obs_ = 2.5 × 10^−4^ s^−1^ (*t*_1/2_ = 0.8 h)) since CICs to MSCs underwent a simple intra-molecule isomerization process.^29,64^

Since the conversion from CICs to MSCs was an intramolecular conversion and the reaction was rapid, we next sought to investigate why CICs would convert to NPLs directly rather than MSCs in our system. Robinson *et al.* showed that the precursor concentrations determined the pathway of MSC formation.^65^ Higher concentrations promoted the formation of MSCs, while lower concentrations inhibited this process. In addition, Robinson also pointed out that when the precursor concentration was below 200 mM, the formation of nanocrystals would dominate at an elevated temperature.^65^ Meanwhile, Yu *et al.* also found that CICs would directly transform into NCs without going through the clustering stage.^29–31^ In our system, the concentration of precursors was less than 0.01 mM. It, therefore, did not favor the formation of MSCs. In addition, higher temperature induced nucleation, while the presence of lamellar mesostructure provided primary-amine-bilayer templates for the domain-limited growth.^36^ Thus, the CICs could be directly converted into flat colloidal nanocrystals (Scheme 1).

Our method is not limited to 3.5 ML CdSe NPLs, but can also be applied to the crystal transformation reaction of thicker NPLs. To illustrate the effect of thickness on the reaction process, 4.5 ML CdSe NPLs were used. We found that in the primary amine system, 4.5 MLs NPL underwent the same phase transition from ZB to WZ as 3.5 ML NPLs. We showed that this transformation process was a ligand-assisted CIC-mediated phase transition pathway, which also involved three steps: Step 1, ligand exchange (Fig. S15a,† black curve); Step 2, formation of CICs (Fig. S15a,† red curve), and Step 3, conversion of CIC assemblies to WZ CdSe NPLs (Fig. S15a,† blue curve). We noted that the first exciton peak of the formed WZ NPLs remained at 450 nm and did not undergo a redshift because, in Step 3, the thickness of the WZ NPL was determined by the reaction temperature. Thin NPLs were formed at lower temperatures (Fig. S15b,† black curve), and thick NPLs at higher temperatures (Fig. S15b,† red curve). The elucidation of Step 1, 2, and 3 provides a comprehensive understanding of the phase transition mechanism in the NPL system and confirms the role of the CIC as intermediates in bridging the phase transition process.

### CIC-assisted phase transition in other NCs

**Fig. 5:**
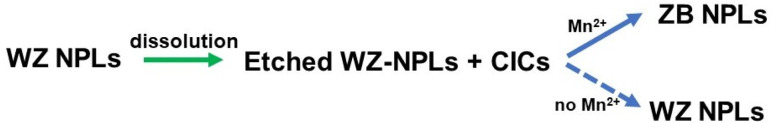
Schematic drawing of the CIC-mediated crystal phase transfer mechanism from WZ NPLs to ZB NPLs (blue arrow) and the CIC-mediated evolution mechanism in the absence of Mn doping (blue dotted arrow).

The transformation from the WZ to the ZB phase in NCs has also been reported during these years. For example, Pradhan *et al.* showed the crystal transformation of 2D ZnS NPLs from WZ to ZB using high-temperature Mn doping.^17,24^ However, the mechanism still needs to be studied. Here, we reevaluated the phase transition process of ZnS NPLs from WZ to ZB and systematically investigated the underlying transition pathway.^24^ Using WZ ZnS NPLs (Fig. S16a†), we observed the crystal phase transition process (from WZ to ZB) by introducing Mn dopants at 300 °C, which was consistent with the literature report (Fig. 5 blue arrow, Fig. S16b†). Compared to the pristine WZ ZnS NPLs, the product at high temperature was ellipsoidal and significantly smaller in size (∼10 nm, Fig. S16c†), indicating that the ZnS NPLs underwent dissolution during the heating treatment. The decline in XRD diffraction quality also provided evidence of NPLs dissolution (Fig. S16c,† inset).

We further investigated the species in the dissolution solution by ESI-MS measurement. We found the existence of covalent complexes ZnS–CICs (Fig. S16e† red line), which subsequently transformed into ZB ZnS NPL upon the introduction of Mn (similar to the Step 2 and 3 described in the phase transition from ZB toWZ of CdSe NPLs, Scheme 1). As expected, the formation of ZB ZnS NPLs was accompanied by the gradual disappearance of ZnS CICs, suggesting that the CICs were intermediates during the crystal phase transition (Fig. S16f†). Subsequently, a control experiment was performed to illustrate the role of Mn in this process. We found that in the absence of Mn doping, ZnS NPLs returned to their original state without crystal structure change (blue dotted arrow in Fig. 5 and S16e† blue line and Fig. S16d†). These results demonstrated that doping with Mn ions could induce a cubic phase reorganization with ZnS–CICs to complete the crystal transformation. This work has provided ideas for a better understanding of the relationship between different crystalline structures and will also provide more insight into the design and synthesis of functional NCs.

## Conclusion

We achieved the first complete crystal phase transformation from ZB to WZ and developed a general ligand-assisted CIC-mediated model (Scheme 1) to describe the phase evolution process in the CdSe NPL system. We have shown that this process involves three distinct steps. The primary amine (L-type) ligand replaced the cadmium oleate (Cd(OA)_2_) through a ligand exchange process (Step 1) on the ZB NPLs, which were further etched from the edge to form covalent complex CdSe–CICs (Step 2). Subsequent assembly and anisotropic growth of the CdSe–CICs allowed the direct formation of WZ CdSe NPLs, with the Cd(OA)_2_ from Step 1 continuing to act as a Z-type ligand to passivate the surface defects (Step 3). Our results provided direct and conclusive experimental evidence for an in-depth insight into the evolution of the NC phase transfer involving the CIC intermediate.

Furthermore, we confirmed that CICs were also present and played an essential role in the crystal phase transition from WZ to ZB NPLs. This work will motivate the exploration of universal nucleation theories and advance our understanding of crystalline systems in other materials. With the current development of the phase transition framework, the synthesis and manipulation of NCs is moving from an empirical art to a science.

## Data availability

The data that support the findings of this study are available from the corresponding author upon reasonable request.

## Author contributions

Y. W. conceived the concept of this work. Y. W. and X. K. designed the experiments, analyzed the data and co-wrote the paper. L. R., J. G. and P. Z. performed optical and ESI measurements. Y. D. performed ICP-OES and TEM measurements. Y. W. supervised the project. All authors discussed the results and commented on the manuscript.

## Conflicts of interest

The authors declare no competing financial interests.

## Supplementary Material

SC-014-D3SC04296K-s001
