# Supplementary material for: Covalent inorganic complexes enabled zinc blende to wurtzite phase changes in CdSe nanoplatelets
Source: Chem Sci. 2023 Nov 8;14(45):13244–53. doi: 10.1039/d3sc04296k (PMC10664457; doi:10.1039/d3sc04296k)
Supplement: SC-014-D3SC04296K-s001 [file SC-014-D3SC04296K-s001.pdf]

Electronic Supplementary Material (ESI) for Chemical Science.
This journal is © The Royal Society of Chemistry 2023

Supporting Information

Covalent Inorganic Complexes Enabled Zinc Blende to Wurtzite Phase Changes in CdSe Nanoplatelets

Xinke Kong,† Lin Ru,† Junjun Ge,† Yalei Deng,† Pan-ke Zhang,† Yuanyuan Wang*, †

† *State Key Laboratory of Coordination Chemistry, State Key Laboratory of Analytical Chemistry for Life Science, School of Chemistry and Chemical Engineering, Nanjing University, Nanjing 210093, China*

* To whom correspondence should be addressed. E-mail: wangyy@nju.edu.cn

EXPERIMENTAL SECTION

Materials. Cadmium acetate dihydrate ($Cd(Ac)_2·2H_2O$, AR, Shanghai Macklin Biochemical Co., Ltd.), *n*-octylamine (99%, Aldrich), butylamine (99%, Aldrich), pentylamine (99%, Aldrich), lauryl amine (99%, Aldrich), oleylamine (99%, Aldrich), 1-octadecene (90%, Aldrich), selenium powder (Se, ≥99.99% metals basis, ≥200 mesh, Aldrich), octadecene (ODE, GC, >90.0%, Aladdin), Triphenylphosphine (TOP, 99%, Aldrich), oleic acid (OA, 90%, Alfa Aesar), hexadecylamine (HDA, Aldrich), sodium diethyldithiocarbamate trihydrate (NaDEDTC, Aldrich), manganese acetate ($Mn(OAc)_2$, 98%, Aldrich) were used as received without further purification unless stated otherwise.

Preparation of ZB CdSe NPLs. ZB CdSe NPLs were synthesized in a nitrogen environment following a previously published method with slight modifications.[1] The Se precursor (TOP-Se, 1 M) was previously prepared in a typical procedure: 79 mg of selenium was dissolved by stirring in a bottle containing 10 mL of TOP in a glove box. In a 50 mL three-necked flask, a mixture of $Cd(OAc)_2$-$2H_2O$ (240 mg), OA (150 µL) and 1-octadecene (15 mL) was stirred and degassed under vacuum (0.2 Torr) at 80 °C for 30 min. The mixture was then heated to 180 °C under a nitrogen stream. When the mixture's temperature reached 180 °C, a syringe containing 160 µL of TOP-Se at 1 M was rapidly injected into the flask. The solution was held at 180 °C for 20 minutes and the reaction was stopped. The temperature was reduced to 80 °C and 2 mL of OA and 10 mL of hexane were added. The mixture was then centrifuged and the precipitate containing the ZB CdSe NPLs was stored in the refrigerator for future use.

Preparation of WZ ZnS NPLs. WZ ZnS NPLs were synthesized following Narayan's published method.[2] Zinc diethyldithiocarbamate (Zn-DEDTC): 1.36 g of zinc chloride was dissolved in 10 ml of methanol and stirred to give a clear solution. This solution was added dropwise with stirring to a methanolic solution of 4.5 g NaDEDTC (in 15 ml methanol) and immediately precipitated to Zn-DEDTC. These precipitates were collected by centrifugation, washed with methanol and dried under vacuum. Manganese diethyldithiocarbamate (Mn-DEDTC): 0.495 g of Na-DEDTC was dissolved in 4 ml of oleylamine and injected at 60 °C into 0.017 g of $Mn(OAc)_2$ (in 4 ml of oleylamine). The mixture slowly turned a pale-yellow color. WZ ZnS NPLs: 3 g of HDA was taken into a 25 ml three-necked flask and purged with nitrogen at 120 °C for 30 min. In a separate flask, 0.1 g of Zn-DEDTC was dissolved in 1 ml of oleylamine and injected into the above reaction flask at 300 °C. The flask was cooled to 280 °C and held for 5 min. Samples were collected at this stage by natural cooling or rapid quenching. Mn-doped ZnS nanoplates: The reaction mixture (WZ ZnS NPLs) was further cooled to 100 °C and injected with 0.2 ml of Mn-DEDTC stock solution. The mixture was then

slowly heated to 300 °C and annealed for 30 min. The sample was collected here via a rapid quenching process.

Transformation of ZB CdSe NPLs to WZ CdSe NPLs. The phase transformation of ZB CdSe NPLs was carried out by the solvothermal method. The appropriate amount of ZB CdSe NPLs (2~4 mg) was redispersed in 3 mL of primary amine solution in a polytetrafluoroethylene reactor. The reaction was kept at 100 °C for about 18 h to complete the transformation. To compensate for the partial loss of surface terminations and obtain more stable crystalline nanoplatelets, the reaction was subjected to in situ thermal annealing at 120 °C for about 2 h. The system was then cooled to room temperature and centrifuged. The resulting precipitate of WZ CdSe NPLs was redispersed in toluene. As an aside, the reaction process was demonstrated in real-time by time sampling at different reaction times at 100 °C.

Supplementary Figures

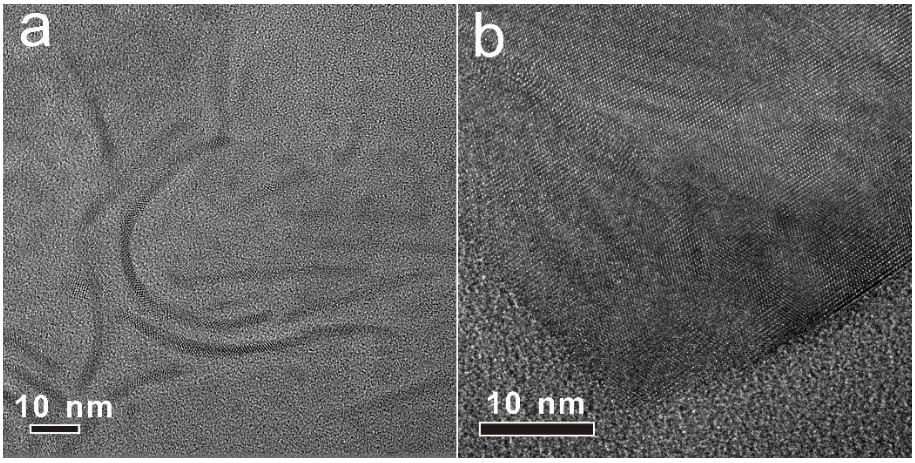

Figure S1. HRTEM images of the original ZB CdSe NPLs (a) and WZ CdSe NPLs (b).

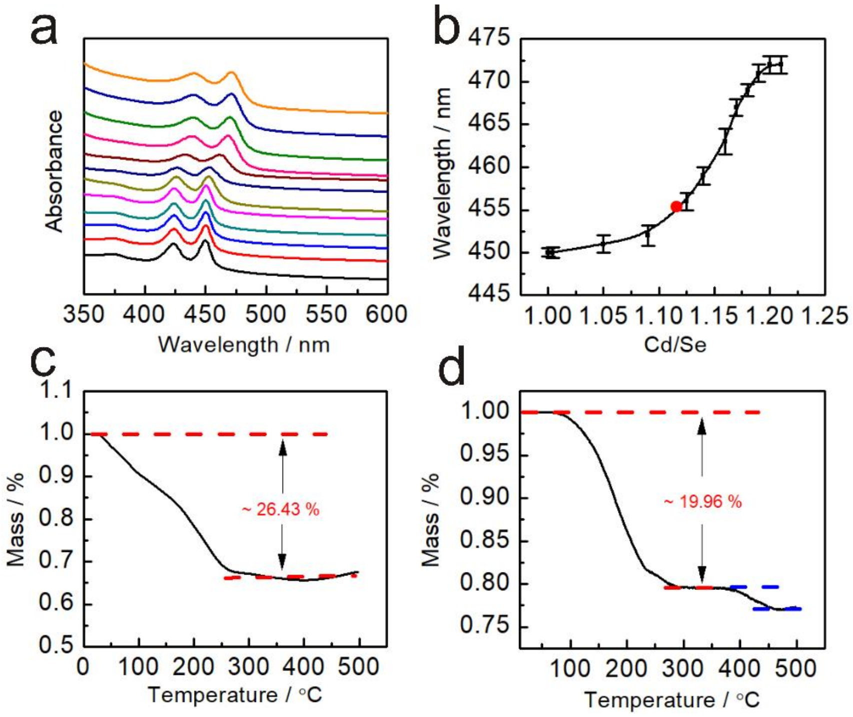

Figure S2. (a) UV-vis absorption spectroscopy of the conventional WZ CdSe NPLs (~ 1 mg) with different amounts of cadmium oleate addition (0 ~ 0.06 mM). (b) Different Cd/Se ratios correspond to the first exciton absorption position (E_1) of the conventional WZ CdSe NPLs with different cadmium oleate adhesion. (c) TGA spectra of the conventional WZ CdSe NPLs (CdSe[octylamine]$_{0.53\pm0.02}$); (d) TGA spectra of the WZ CdSe NPLs obtained from the phase conversion of ZB CdSe NPLs.

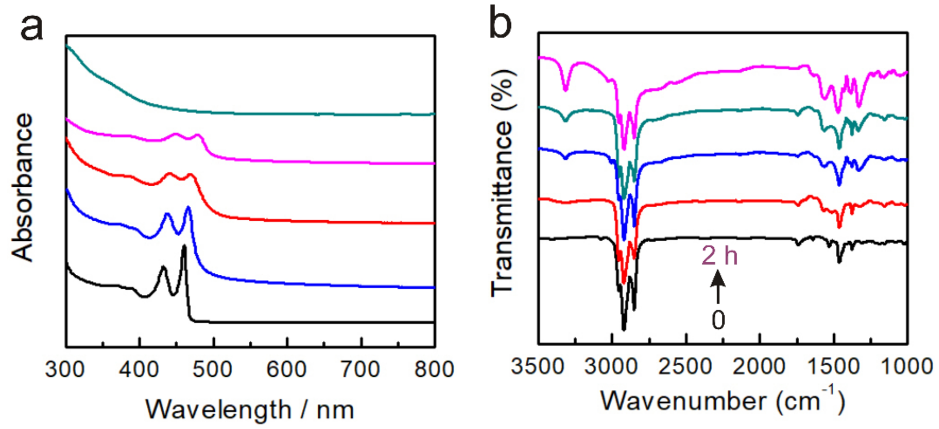

Figure S3. (a) Temporal evolution of the UV-vis absorption of ZB CdSe NPLs with intermittent sampling from the reaction at 100 °C for different times (0~2 h, 0.5 h interval from bottom to top). (b) FT-IR spectroscopies of ZB CdSe NPLs with intermittent sampling (0~2 h, 0.5 h interval from bottom to top) from the reaction at 60 °C.

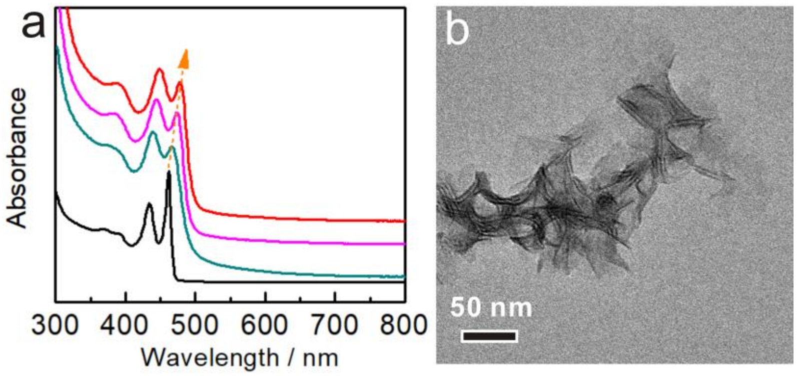

Figure S4. (a) UV–vis absorption spectrum of ZB CdSe NPLs (black: original NPLs) incubated with octylamine for different times (0 ~ 12 h) at 25 °C. (b) TEM image of ZB CdSe NPLs after ligand exchange with octylamine at 25 °C (corresponding to the redline of Figure S4a).

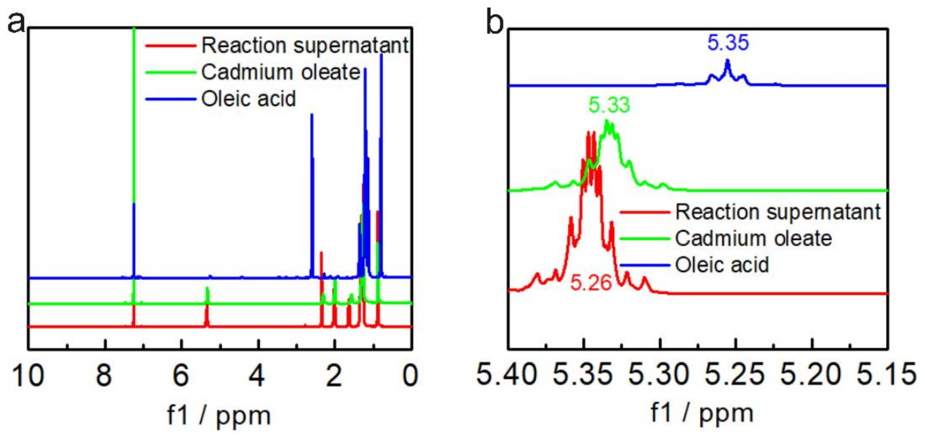

Figure S5. (a) ^{1}H NMR spectrum of purified oleic acid (blue), cadmium oleate (green), and the supernatant of ZB CdSe NPLs after exchange. (b) The vinyl region of the ^{1}H NMR spectrum of samples shows displacement of cadmium oleate after treatment with octylamine.

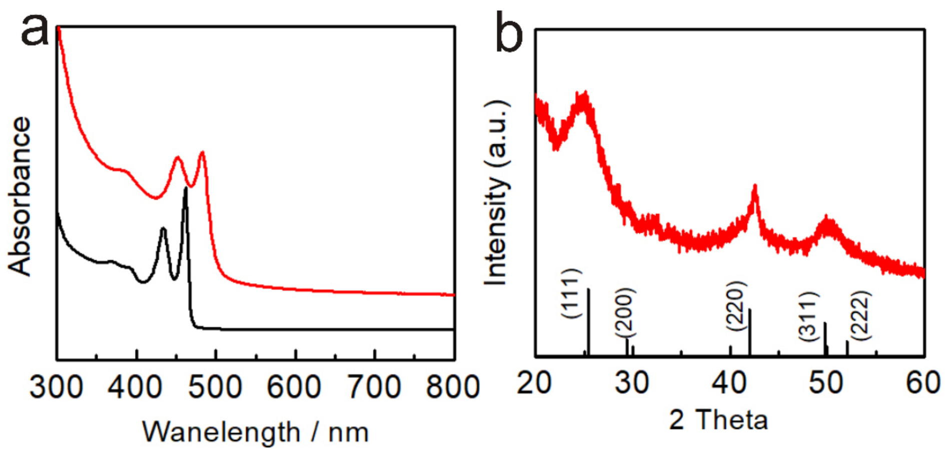

Figure S6. (a) UV–vis absorption spectrum of ZB CdSe NPLs (black: original NPLs) incubated in octylamine/ODE (v:v = 1:2) at 100 ℃ (red). (b) XRD pattern of ZB CdSe NPLs after incubation in octylamine/ODE (v:v = 1:2).

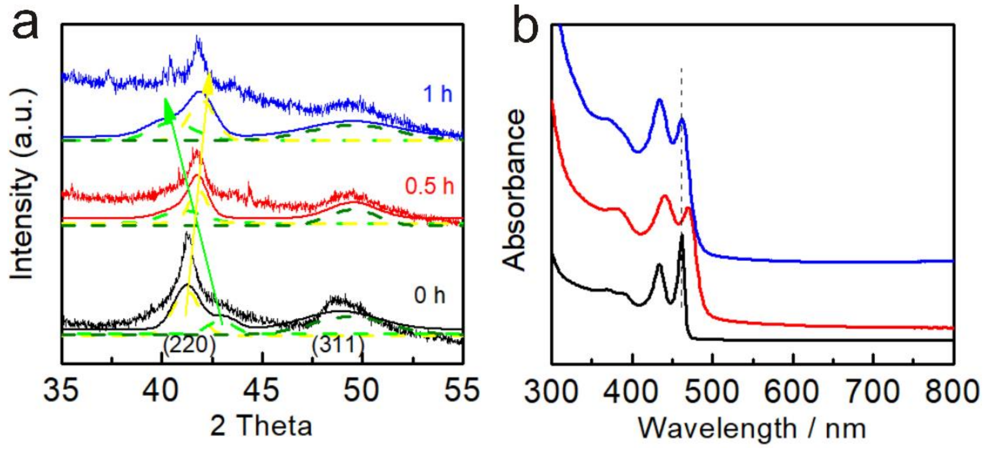

Figure S7. (a) XRD patterns and fitting curves (solid lines) of ZB CdSe NPLs with the same intermittent sampling in octylamine incubation. The (220) crystal plane diffraction of ZB CdSe NPLs contains two diffraction peaks, which were divided into the side (yellow) and thickness (green) directions. The lateral diffraction angle increased (yellow dotted line), while the thickness direction diffraction angle decreased and widened (green dotted line) after incubation in octylamine liquor with ligand exchange. (b) UV–vis absorption spectrum (black: original NPLs; red: NPLs incubated with octylamine; blue: back exchange with $Cd(OA)_2$) of ZB CdSe NPLs in toluene.

Table S1. The lattice parameter information of NPLs for (220) according to the XRD patterns in Figure S7 (nm).

Samples	$d_{220,\ lateral}$	$d_{220,\ thickness}$	a	c	a/c
0 h	0.219	0.211	0.619	0.697	1.041
0.5 h	0.216	0.219	0.610	0.620	0.984
1 h	0.215	0.222	0.608	0.628	0.968

Energy shift calculation[3, 4]:

$$\Delta E = -\alpha(c_x - c_{OA})Y/c_{OA} \qquad \text{(Equation S1)}$$

α: Band gap pressure coefficient 43.1 meV/Gpa.

Y: Young's modulus 48.9 GPa.

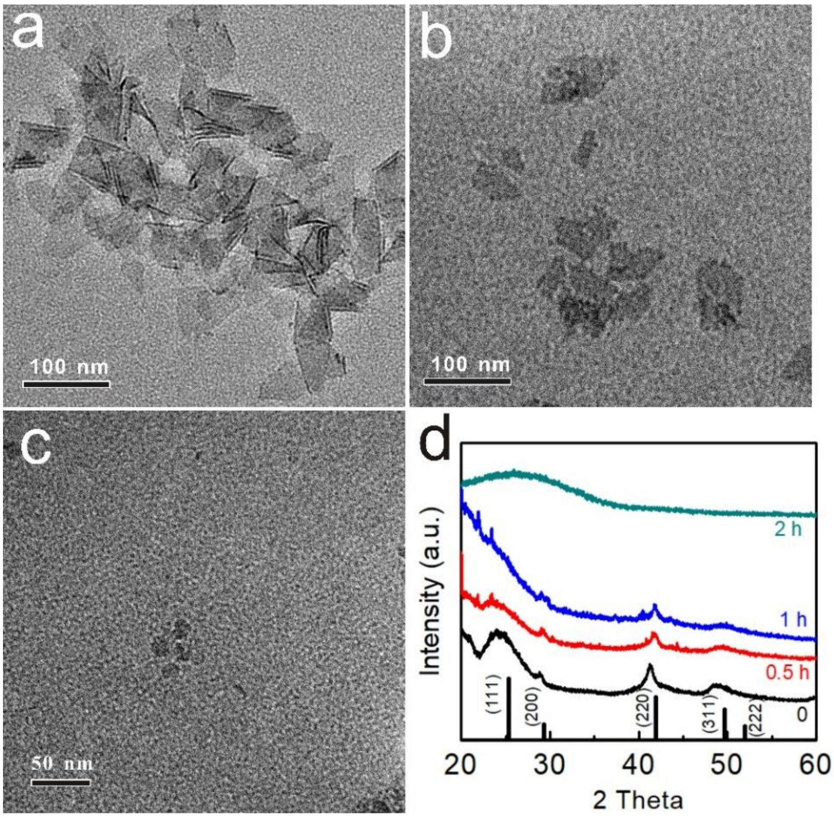

Figure S8. (a, b, c) TEM images of ZB CdSe NPLs with intermittent sampling (0.5 h, 1 h, 2 h). (d) Temporal evolution of the XRD patterns of ZB CdSe NPLs with the same intermittent sampling.

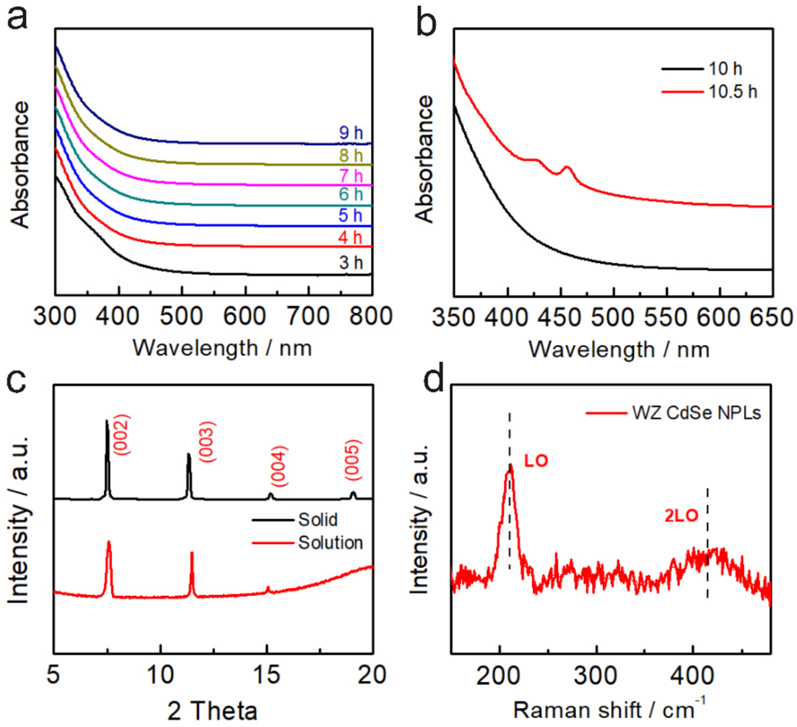

Figure S9. (a, b) UV-vis absorption spectra collected from the reaction with intermittent sampling. (c) XRD spectra of CdSe-CIC (date from solid state (black) and solution (red)). (d) Raman spectra of the obtained WZ CdSe NPLs.

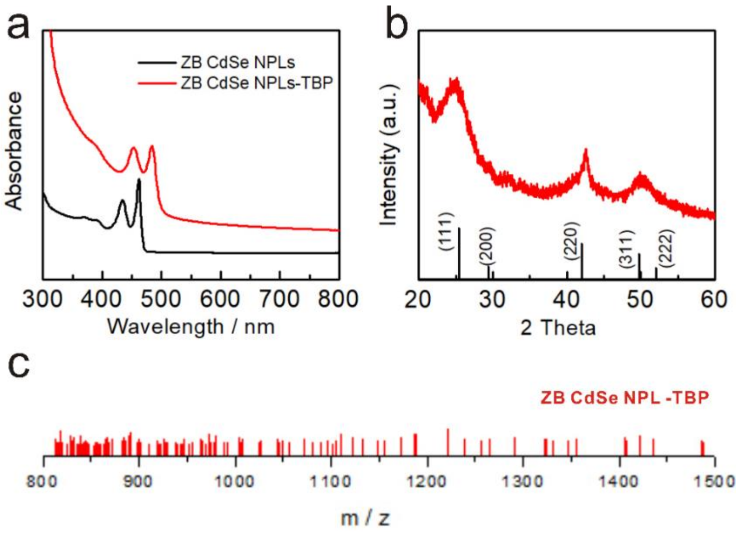

Figure S10. (a) UV-vis absorption spectra of ZB CdSe NPLs before (black line) and after (red line) incubation with TBP. (b) XRD pattern of CdSe NPLs after TBP incubation. (c) ESI-MS spectra of the sample extracted from the reaction of ZB CdSe NPLs with TBP in the m/z range from 800 to 1500 Da.

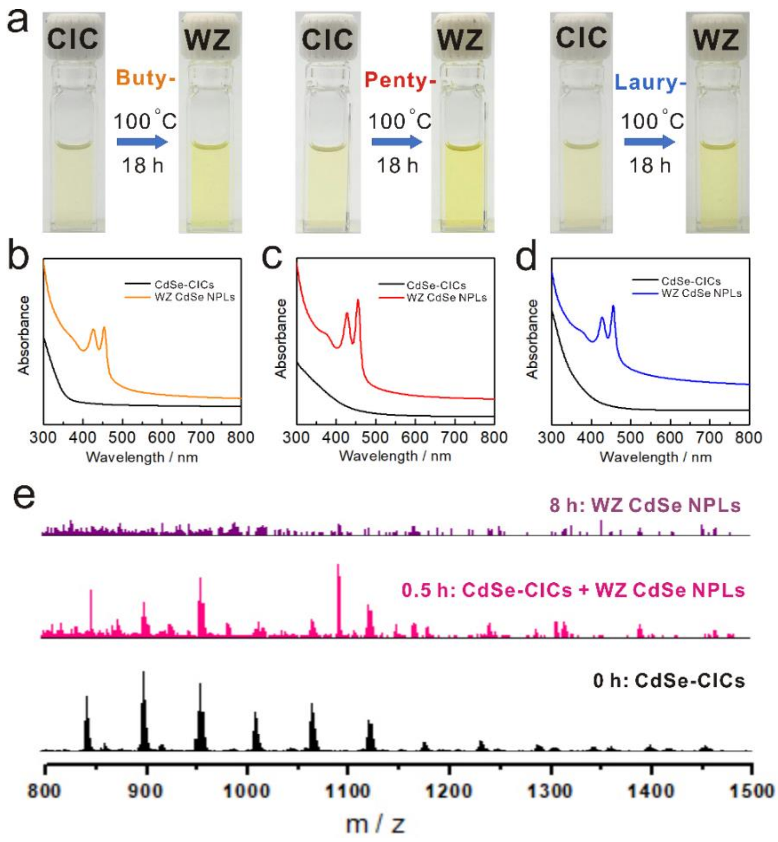

Figure S11. Relevant experimental results for the phase transformation of ZB CdSe NPLs to CIC to WZ CdSe NPLs in butylamine, pentylamine, and laurylamine systems. (a) Reaction condition and photograph of CdSe-CICs and product WZ CdSe NPLs in butylamine (left), pentylamine (center), and laurylamine (right) systems. (b, c, d) UV-vis absorption spectra of CdSe-CICs and product WZ CdSe NPLs in the three systems. (e) Temporal evolution of the ESI-MS spectrum in the octylamine system with different sampling times according to Figure 4a.

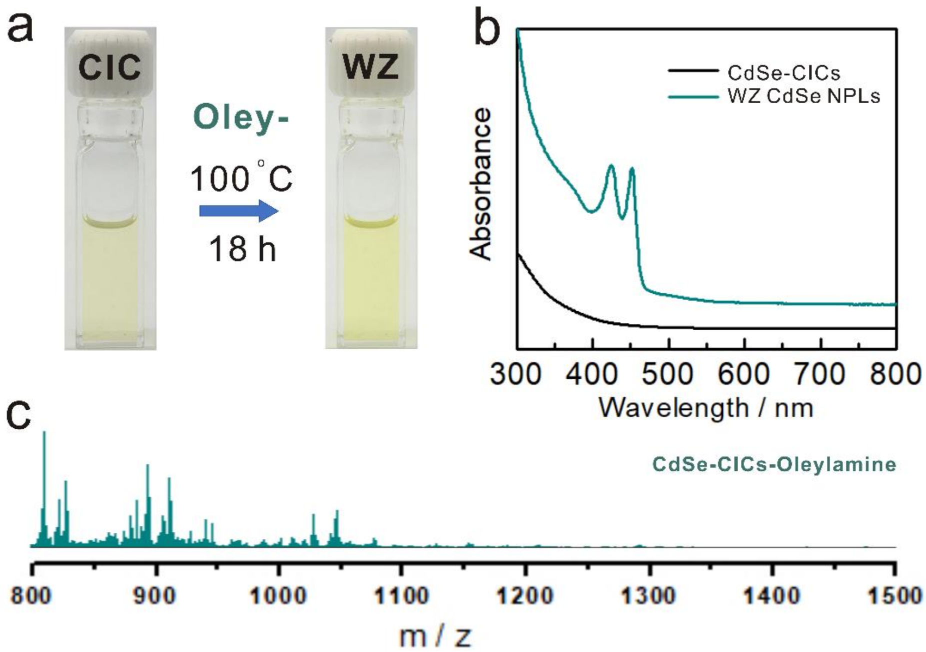

Figure S12. (a) Reaction condition and photograph of CdSe-CICs and product WZ CdSe NPLs in oleylamine system. (b) UV-vis absorption spectra of CdSe-CICs and the product WZ CdSe NPLs in an oleylamine system. (c) ESI-MS spectra of the intermediate CdSe-CICs under the oleylamine system in the m/z range from 800 to 1500 Da.

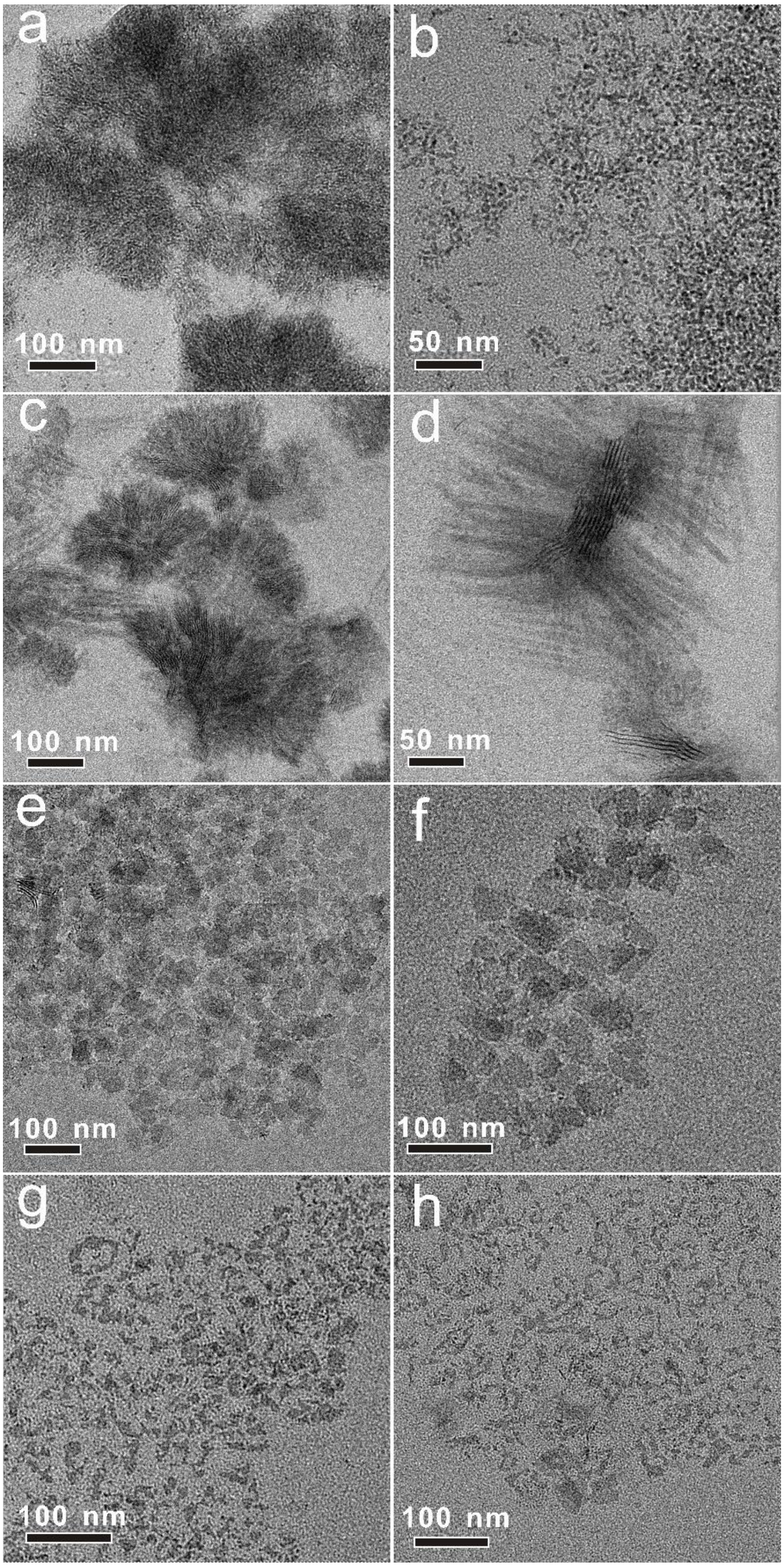

Figure S13. TEM images of WZ CdSe NPLs after the transformation from ZB CdSe NPLs in (a, b) butylamine, (c, d) pentylamine, (e, f) laurylamine and (g, h) oleylamine systems.

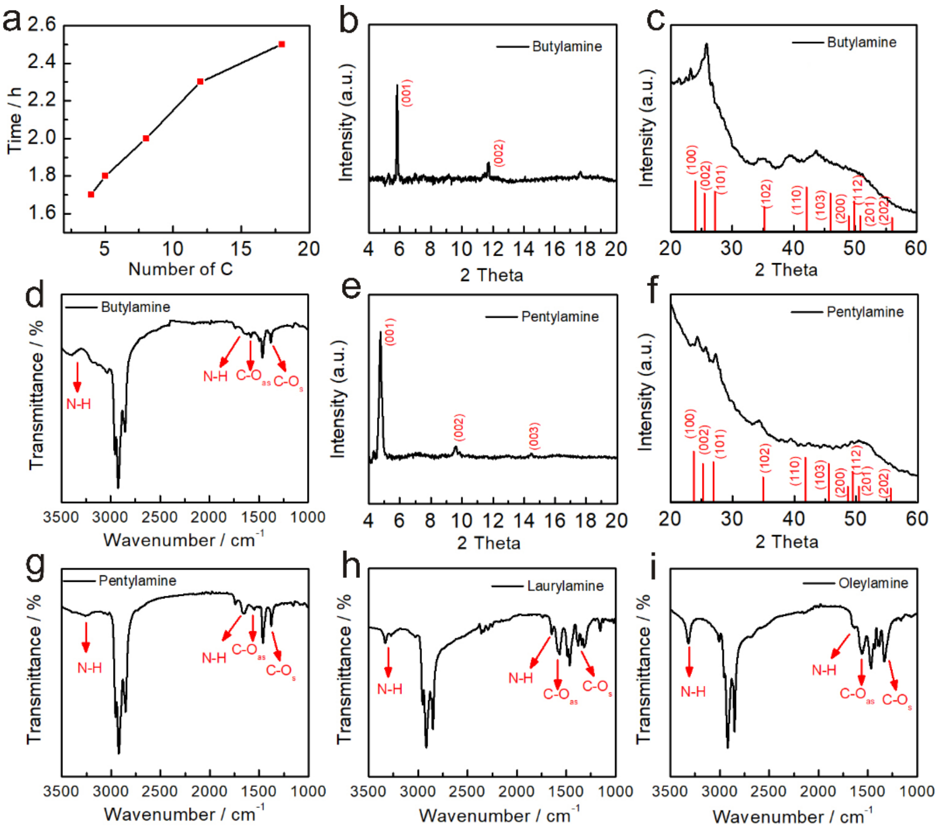

Figure S14. Mainly crystal morphology data of CdSe NPLs after conversion in butylamine, pentylamine, laurylamine, and oleylamine systems. (a) The correlation curve for primary amine carbon chain lengths with the time of complete disappearance of NPL absorption. XRD patterns and IR spectra of the post-transformation product WZ CdSe NPLs in butylamine (b, c, d) and pentylamine (e, f, g). IR spectra of the product WZ CdSe NPLs in laurylamine (h) and oleylamine (i).

To illustrate the role of primary amine ligands in the crystal transformation, ZB CdSe NPLs were incubated in various primary amines with different carbon chain lengths (C_4 to C_{18}). As expected, the high-angle XRD features of the products (**Figure S14c** and **S14f**) showed the standard WZ hexagonal unit cell diffraction. The two-dimensional structure absorption features (**Figure S11b~d** and **Figure S12b**) were at 453/425 nm (*n*-butylamine), 455/427 nm (*n*-pentylamine), 455/427 nm (*n*-lauramine) and 453/425 nm (*n*-oleylamine), which were consistent with those of the NPLs obtained from *n*-octylamine (**Figure 1b, red curve**). The slight variations in the positions (1~3 nm) and shapes of the absorption spectra between products were caused by small perturbations in the surface or electronic structure due to changes in the surface ligands.[5, 6] TEM images showed sheet-like two-dimensional morphologies with slight differences in the lateral dimension (**Figure S13**). Corresponding low-angle XRD evidenced lamellar NPL assemblies with different interlayer spacings *d* (**Figure S14b** and **S14e**). Further characterization of the surface states by FT-IR (**Figure S14d and S14g~i**) and ICP-OES analyses (higher Cd/Se ratios 1.08 to 1.15) were consistent with the product in the

octylamine system in the previous section. However, it is worth mentioning that different amine systems have different reaction rates. At the same temperature (100 °C), the shorter the chain length of the primary amine, the faster the reaction (**Figure S14a**), which could be due to the difference in the boiling point of the different chain lengths of the primary amines.

In addition, the ZB CdSe NPLs in the various primary amine systems all dissolved to form a transparent solution with no characteristic optical absorption (**Figure S11a-d**, **Figure S12a-b**). Further determination by ESI-MS indicated the formation of CdSe-CICs (**Figure S11e**, **Figure S12c**). We found that the atomic composition distribution of CICs varied in different primary amines, possibly due to the stability of the CICs in corresponding amines. Notably, the different atomic compositions of the CdSe-CICs did not affect on their conversion in primary amines to two-dimensional products, and no other intermediates were detected during the transformation. These results showed that the NPL crystal phase transition can occur in different primary amine systems with the same evolutionary mechanism.

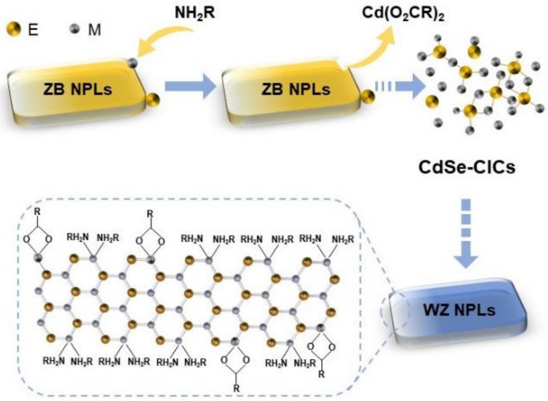

Scheme S1. Schematic of the evolutionary mechanism of ligand action for the primary amine-driven phase transfer from ZB CdSe NPLs to WZ CdSe NPLs.

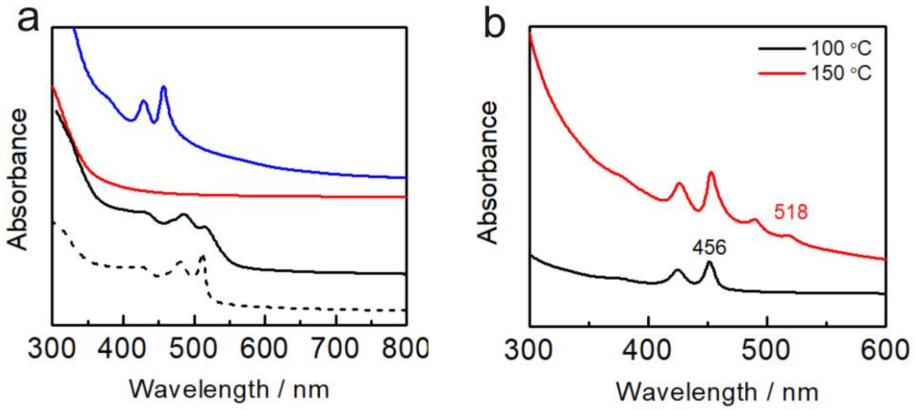

Figure S15. UV-vis absorption spectra (a) of ZB 4ML CdSe NPLs (black dotted line) after incubation in octylamine at 100 °C for 1 h (black), 6 h (red) and 24 h (blue). UV-vis absorption

spectra (b) of products WZ CdSe NPLs after incubation in octylamine at 100 °C (black) and 150 °C (red).

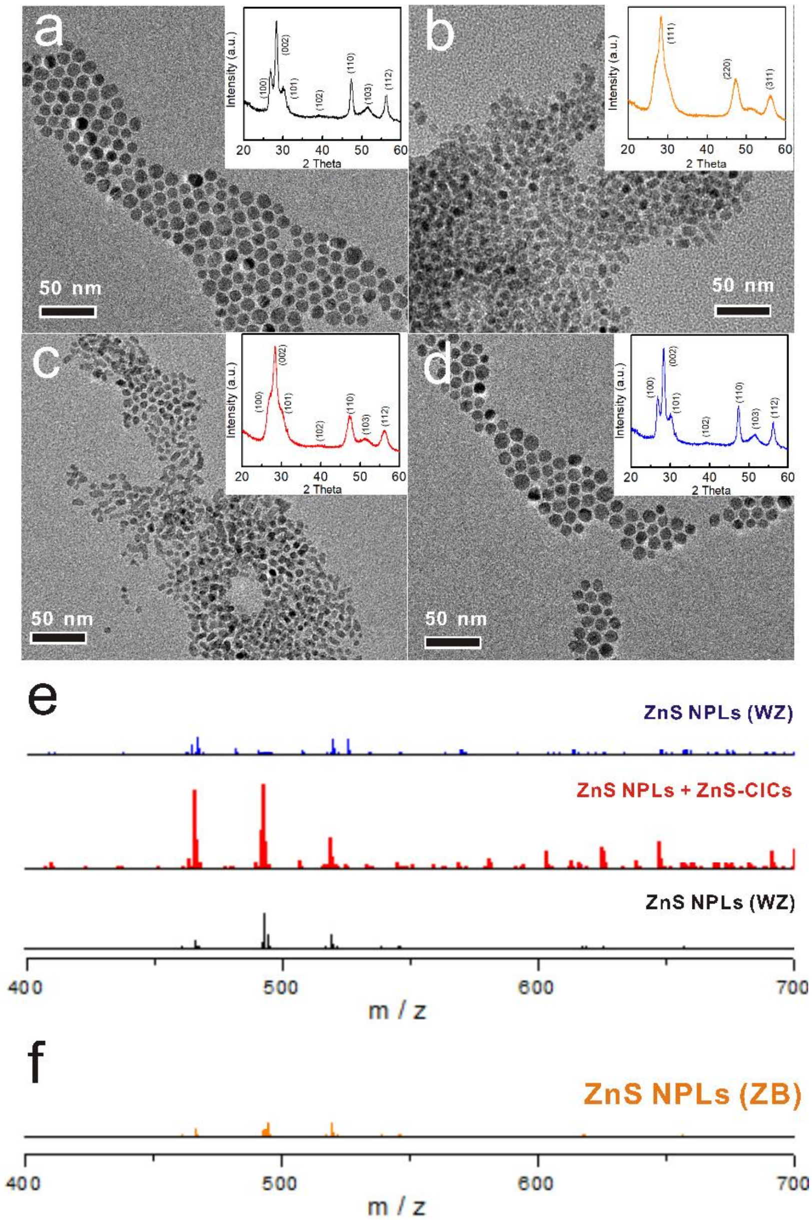

Figure S16. Relevant experimental results for the ZnS NPL system. TEM and XRD characterizations of original WZ ZnS NPLs (a) and Mn-doped ZnS NPLs after annealing at 300 °C with a fast quenching (b). TEM and XRD characterizations of WZ ZnS NPLs (a) after annealing at 300 °C without Mn addition with fast quenching (c) or slow cooling (d). ESI-MS spectra of the supernatant of the systems corresponding to the original WZ ZnS NPLs (black), after annealing at 300 °C with a fast quenching (red) or slow cooling (blue) (e). (f) ESI-MS spectra of the supernatant of the sample corresponding to b.

References

[1] C. She, I. Fedin, D. S. Dolzhnikov, P. D. Dahlberg, G. S. Engel, R. D. Schaller and D. V. Talapin, *ACS Nano* **2015**, *9*, 9475-9485.
[2] N. S. Karan, S. Sarkar, D. D. Sarma, P. Kundu, N. Ravishankar and N. Pradhan, *J. Am. Chem. Soc.* **2011**, *133*, 1666-1669.
[3] Y. Zhou, F. Wang and W. E. Buhro, *J. Am. Chem. Soc.* **2015**, *137*, 15198-15208.
[4] B. T. Diroll and R. D. Schaller, *Chem. Mater.* **2019**, *31*, 3556-3563.
[5] Y. Deng, J. Liang, X. Kong, P. Xiao, Y. Zhou and Y. Wang, *Chem. Mater.* **2023**, *35*, 2463-2471.
[6] B. M. Cossairt, P. Juhas, S. Billinge and J. S. Owen, *J. Phys. Chem. Lett.* **2011**, *2*, 3075-3080.
